# Forensic facial approximation methodologies applied to the Brazilian population: a scoping review

**DOI:** 10.1007/s00414-026-03947-2

**Published:** 2026-08-08

**Authors:** Paula de Sousa Rachid, Daniel Pignatari Mahet Rodrigues, Lucas Alves Jural, Andrea Vaz Braga Pintor, Lucianne Cople Maia, Antônio Carlos de Oliveira Ruellas, Maria Augusta Visconti, Andreia Cristina Breda de Souza

**Affiliations:** 1 School of Dentistry, Universidade Federal do Rio de Janeiro., Rio de Janeiro, RJ Brazil; 2 School of Dentistry, Universidade Federal de Uberlândia, Uberlândia, MG Brazil; 3 School of Dentistry, Universidade Federal Fluminense, Niterói, RJ Brazil

**Keywords:** Forensic facial approximation, Forensic facial reconstruction, Facial soft tissue thickness, Scoping review

## Abstract

This scoping review aimed to map forensic facial approximation (FFA) methodologies applied to the Brazilian population. The protocol was registered in the Open Science Framework and adopted the following structure: Population, human; Concept, FFA methodologies; and Context, studies conducted in the Brazilian population. Searches were conducted in seven databases and grey literature up to December 2025. No restrictions were applied regarding publication date or language. The data collected included FFA methodology, sample size, sex, age, region of the country, skull acquisition method, reconstructed facial region, anatomical landmarks, cranial analysis, and facial reconstruction software. Thirty studies proposing new or adapted methodologies were included, totaling 3,239 evaluated samples, predominantly from adult populations and Southeast Brazil (83.3%). Craniofacial structures were obtained mainly through cone-beam computed tomography and facial reconstructions or assessment of facial soft tissue thickness (FSTT). Of these, 23 focused on FSTT and the inclusion of cutaneous appendages, while 7 investigated the dimensions and projection of the nose, mouth, and ears. The studies also examined variations according to sex, age, skeletal class, skin color, and nutritional status. Population biases and the persistence of few studies on the nose, eyes, mouth, and ears remain central challenges, as does the standardization of landmarks and the validation of FSTT tables. Thus, it is observed that the growth of the country’s scientific output in FFA does not yet eliminate critical methodological gaps.

**Supplementary Information:**

The online version contains supplementary material available at https://doi.org/10.1007/s00414-026-03947-2.

## Introduction

Forensic facial approximation (FFA) is an auxiliary identification method that seeks to reproduce the likely facial appearance of an individual based on cranial morphology, facilitating recognition by relatives or acquaintances and supporting subsequent identification processes [1–3]. Beyond facial soft tissue thickness (FSTT), five fundamental principles have been established to guide facial reconstruction: the relationship between the eye and the orbit, the morphology of the nasal tip, the position of the ears, the width of the mouth, and the length of the ears [1]. These principles underscore the wide range of studies required to accurately simulate facial features based on cranial anatomy.

In Brazil, facial reconstruction has frequently been applied in historical and archaeological contexts, including reconstructions of archaeological remains, saints, and historical figures [4, 5]. Such applications often combine scientific methodology with cultural and social interpretations of cranial remains. In contrast, FFA prioritizes minimal subjectivity and high precision, as its main objective is to facilitate recognition and enable subsequent human identification [6], and has been used to assist in the identification of corpses in the country [7].

In Brazil, FFA proves to be an important method to assist in the identification of some of the more than 80,000 missing persons records per year [8], and the hundreds of unidentified corpses accumulated in forensic banks and medico-legal institutes [9–11]. Therefore, estimating facial dimensions with reduced subjectivity directly contributes to the generation of investigative hypotheses.

The FFA process typically begins with an anthropological assessment of the skull, followed by the reconstruction of facial soft tissues and anatomical structures. This process is influenced by factors such as ancestry, sex, age, and nutritional status. Facial features can be reconstructed using the skull itself, medical imaging techniques such as radiography and computed tomography (CT), plaster casts, or three-dimensional (3D)-printed models. Depending on the available materials and methodological approach, reconstructions may be performed in two-dimensional (2D) or 3D formats, using either digital or manual techniques [1].

Historically, FFA has been based on three principal methodological approaches worldwide: the Russian or anatomical method, which reconstructs facial musculature individually; the American or anthropometric method, which relies on population-based average measurements of FSTT; and the Manchester or combined method, which integrates muscular modeling with soft tissue depth data [1].

Given that FFA is an important tool for identifying human remains [1–3], the absence of biometric standards specifically developed for the Brazilian population, together with reliance on methodologies derived from other populations, highlights the relevance of this research [1]. Therefore, the present study aims to identify, map, and synthesize FFA methodologies applied to the Brazilian population, examining variables such as sex, age, sample source, techniques employed, and selected anatomical reference points. By doing so, this study seeks to consolidate the existing body of knowledge, identify methodological gaps, and provide a foundation for the development of more accurate national protocols.

## Materials and methods

### Protocol and registration

This scoping review was conducted in accordance with the methodology proposed by the Joanna Briggs Institute (JBI) [12] and reported following the Preferred Reporting Items for Systematic Reviews and Meta-Analyses Extension for Scoping Reviews (PRISMA-ScR) guidelines [13]. The review was guided by the following research question: *“Which methodologies for forensic facial approximation have been applied to Brazilian human populations?”* The review protocol was registered in the Open Science Framework (OSF) and is available at: https://osf.io/ycr95/.

## Eligibility criteria

Eligibility criteria were defined according to the Participant, Concept, and Context (PCC) framework recommended by the JBI. The population (P) comprised human populations; the concept (C) referred to methodological approaches to FFA; and the context (C) included studies conducted in Brazil. Full-text scientific articles describing primary studies that proposed new or adapted FFA methodologies applied to Brazilian populations were included, regardless of skin color, sex, age, nutritional status, facial typology, or skeletal class.

Exclusion criteria included studies that did not describe the methodology employed (inconsistent concept) and studies that applied the methodology to archaeological or fossil human populations (ineligible participants). Additionally, review articles, dissertations, theses, opinion papers, letters, protocols, and editorials were excluded.

## Information sources and search strategy

Preliminary searches were performed in PubMed to identify relevant keywords in article titles and abstracts, as well as controlled vocabulary terms used for indexing studies related to the topic. Subsequently, searches were conducted in PubMed using terms from the Health Sciences Descriptors (DeCS/MeSH), associated entry terms, and free-text terms, from which the initial search strategy was developed and tested. This strategy was then adapted for the remaining databases, Scopus, Embase, LILACS, VHL, Web of Science, and Cochrane, according to the specific syntax requirements of each database.

In addition, grey literature was searched using Google Scholar and OpenGrey. The first 200 records retrieved from Google Scholar were analyzed for potential eligibility. Database searches were conducted and independently evaluated by two blinded reviewers (PSR and DPMR) in May 2025. From this date, update alerts were set up for all databases and subsequently reviewed in December 2025. Finally, the reference lists of the included studies were examined to identify additional relevant publications.

## Study selection

All identified records were imported into the EndNote Web^®^ reference management software, where duplicates were automatically removed. The remaining records were subsequently exported to the Rayyan^®^ platform for manual duplicate removal and screening. The screening process was conducted independently by two reviewers (PSR and DPMR), who assessed titles and abstracts according to the predefined eligibility criteria. Any discrepancies were resolved through discussion, and when necessary, a third senior reviewer (ACBS) was consulted to reach consensus. The full texts of potentially eligible studies were then assessed to confirm their inclusion. Studies published in languages not known to the reviewers were translated using Google Translate (https://translate.google.com.br/*).* After automatic translation, the technical terms were checked by experts or via technical dictionaries (e.g., Black’s Law or glossaries of anthropology). The search strategies used for each database are presented in Table 1.

**Table 1:** Search strategy for the electronic databases assessed (May 13th, 2025 and updated through databases alerts up to December 2nd, 2025)

DATABASE	SEARCH KEYS
**PubMed** (https://www.ncbi.nlm.nih.gov/sites/pubmed)	(Forensic Sciences[MeSH Terms] OR Forensic Sciences[Title/Abstract] OR Forensic facial reconstruction[Title/Abstract] OR Forensic facial approximation[Title/Abstract] OR Facial soft-tissue thickness[Title/Abstract] OR physiognomic reconstruction[Title/Abstract] OR Forensic Anthropology[MeSH Terms] OR Forensic Anthropology[Title/Abstract] OR Human Identification[Title/Abstract] OR Human Identifications[Title/Abstract]) AND (Brazil[MeSH Terms] OR Brazil[Title/Abstract] OR Brazilian[Title/Abstract] OR Brazilians[Title/Abstract]) AND (Humans[MeSH Terms] OR Humans[Title/Abstract])
**Scopus** (https://www.scopus.com)	INDEXTERMS (“Forensic Sciences" OR "Forensic Anthropology”) OR TITLE-ABS-KEY (“Forensic Sciences" OR "Forensic facial reconstruction" OR "Forensic facial approximation" OR "Facial soft-tissue thickness" OR "physiognomic reconstruction" OR "Forensic Anthropology" OR "Human Identification" OR "Human identifications”) AND INDEXTERMS (“brazil”) OR TITLE-ABS-KEY (brazil*) AND INDEXTERMS (“humans”) OR TITLE-ABS-KEY (human*)
**Embase** (https://www.embase.com)	(‘forensic science’/exp OR ‘forensic anthropology’/exp OR ‘forensic sciences’:ti, ab, kw OR ‘forensic science’:ti, ab, kw OR ‘forensic facial reconstruction’:ti, ab, kw OR ‘forensic facial approximation’:ti, ab, kw OR ‘facial soft-tissue thickness’:ti, ab, kw OR ‘physiognomic reconstruction’:ti, ab, kw OR ‘forensic anthropology’:ti, ab, kw OR ‘human identification’:ti, ab, kw OR ‘human identifications’:ti, ab, kw) AND (‘brazil’/exp OR ‘brazil*’:ti, ab, kw) AND (‘human’/exp OR human*)
**Lilacs** (VHL – https://bvsalud.org)	(mh: (forensic sciences OR forensic anthropology)) OR (forensic sciences OR forensic facial reconstruction OR forensic facial approximation OR facial soft-tissue thickness OR physiognomic reconstruction OR forensic anthropology OR human identification OR human identifications) AND (mh: (brazil)) OR (brazil OR brazilian OR brazilians) AND (mh: (humans)) OR (humans) AND db: (“LILACS” OR “BBO”) AND instance:“regional”
**Web of Science** (https://webofknowledge.com)	((TS=(“Forensic Sciences” OR “Forensic facial reconstruction” OR “Forensic facial approximation” OR “Facial soft-tissue thickness” OR “physiognomic reconstruction” OR “Forensic Anthropology” OR “Human Identification” OR “Human Identifications”)) AND TS=(Humans)) AND TS=(Brazil OR Brazilian OR Brazilians)
**Cochrane** (https://cochranelibrary.com)	IDSearchHits #1MeSH descriptor: [Forensic Sciences] explode all trees #2MeSH descriptor: [Forensic Anthropology] explode all trees #3"Forensic Sciences” OR “Forensic facial reconstruction” OR “Forensic facial approximation” OR “Facial soft-tissue thickness” OR “physiognomic reconstruction” OR “Forensic Anthropology” OR “Human Identification” OR “Human Identifications” #4#1 OR #2 OR #3 #5MeSH descriptor: [Humans] explode all trees #6Humans900832 #7#5 OR #6,900,835 #8MeSH descriptor: [Brazil] explode all trees #9Brazil OR Brazilian OR Brazilians #10#8 OR #9 #11#4 AND #7 AND #10
**Google Scholar** (https://scholar.google.com.br/)	Forensic Sciences OR Forensic facial reconstruction OR Forensic facial approximation) AND (Brazil OR Brazilian OR Brazilians) AND Humans
**DANS** (https://lifesciences.datastations.nl/)	(“forensic facial approximation” OR “forensic facial reconstruction”) AND (brazil OR brazilians)

## Data mapping and analysis process

Two reviewers (PSR and DPMR) independently conducted the data extraction and mapping process using Google Docs spreadsheets previously developed and pilot-tested for this purpose. The following information was extracted from the included studies: basic descriptive data (authors and year of publication); study design; study objective; sample size, sex, age, and region of the country; whether comparisons were made between the Brazilian population and populations from other countries; method of skull acquisition; reconstructed facial region; theoretical approach applied (British, Russian, or American); number and anatomical reference points used; cranial analysis and facial reconstruction software; number of reconstruction operators; presence of prior training; reported Kappa/ICC reliability scores; and whether reconstruction evaluators were involved.

Additionally, data on analytical methods, the main results on FSTT or measured distances, authors’ conclusions, funding sources, potential conflicts of interest, and other findings relevant to the review question were extracted. Extracted data are presented in data extraction tables, while graphs and figures were created by the author using Canva Pro. To map the available evidence regarding existing methodologies, studies reconstructing the same anatomical regions were grouped, and a descriptive qualitative synthesis of the reported data was performed. Furthermore, the main methodological characteristics of studies that used FSTT results were compiled.

## Results

**Fig. 1:**
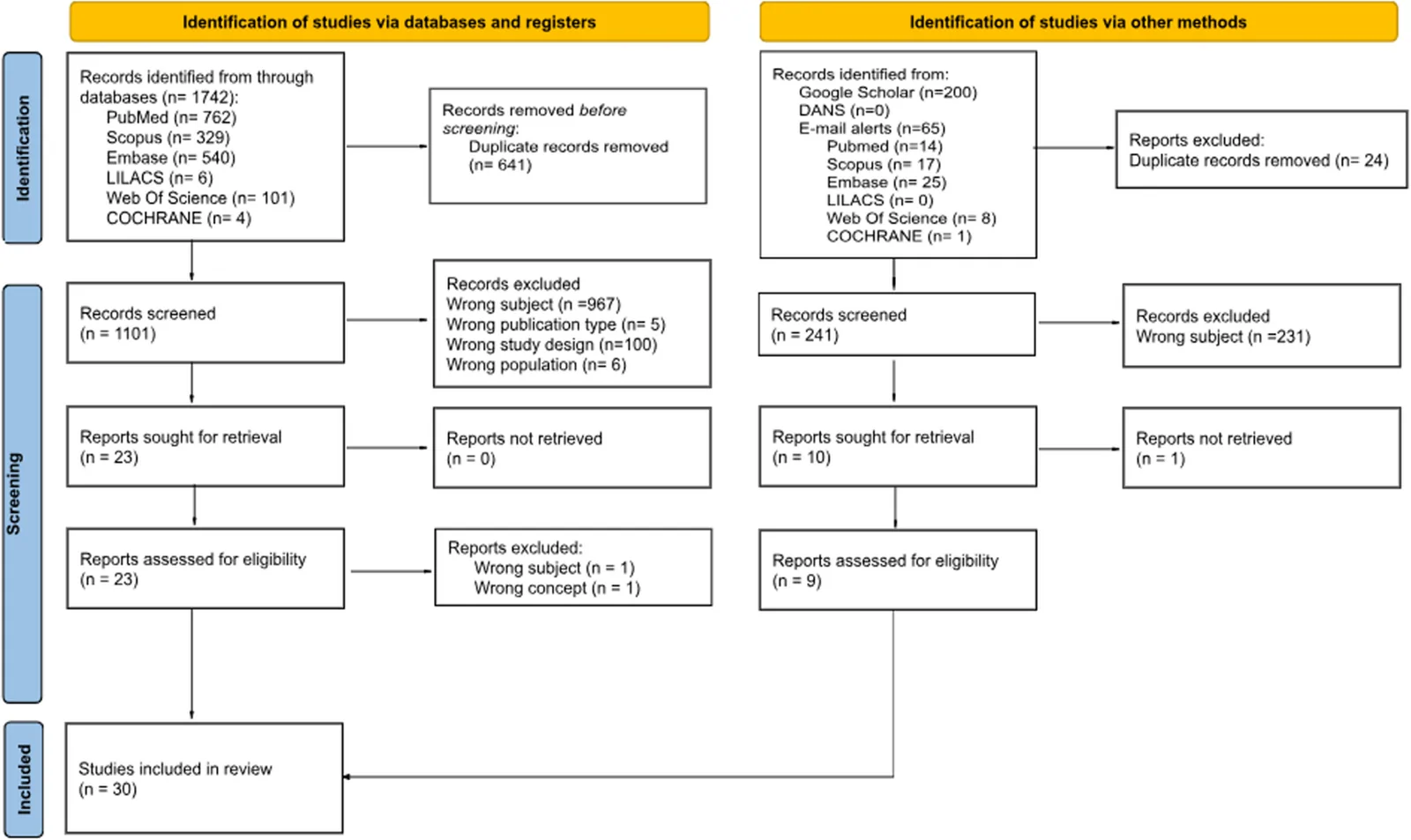
PRISMA 2020 flow diagram for new systematic reviews which included searches of databases, registers and other sources

The initial search retrieved 1,742 records from the databases consulted. After duplicate removal, 1,101 records were screened based on titles and abstracts. Of these, 23 studies were selected for full-text assessment, and 2 were subsequently excluded (Online Resource 1). Following the grey literature search and evaluation of database update alerts, nine additional eligible studies were identified. In total, 30 studies [14–43] were included in the present review (Fig. 1).

## Extracted data

### General data

**Fig. 2:**
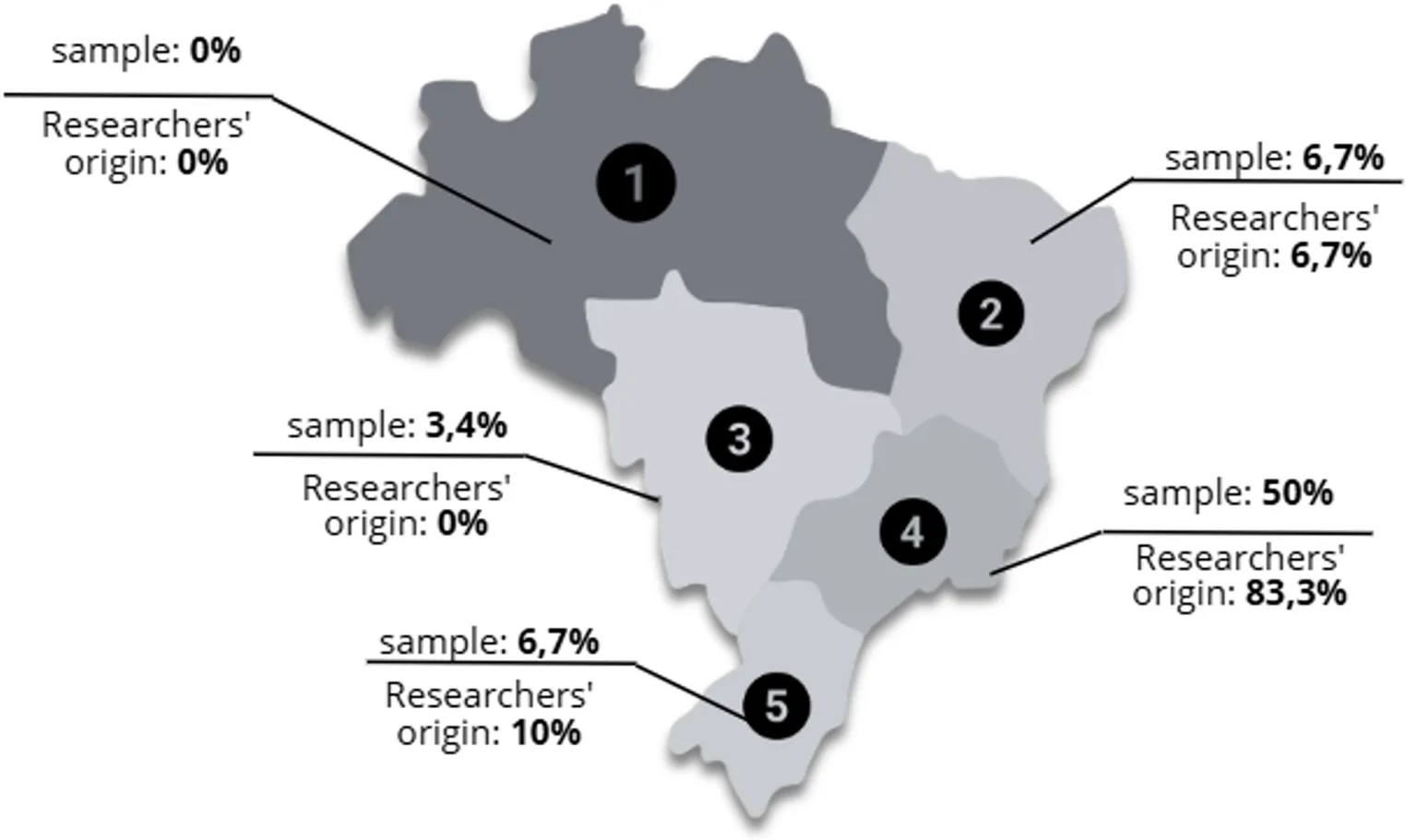
Geographic distribution of the cited samples and origin of the included studies by Brazilian regions. Legend: Geographical regions of the country: 1- North; 2- Northeast; 3- Central-West; 4- Southeast; 5- South

Extracted data were categorized into groups, including general study information (Table 2), population characteristics, methodological details, reported results, and study conclusions (Online Resource 2). The included studies were published between 2003 and 2024, with most publications occurring after 2015. In all studies, the first author was Brazilian and was predominantly affiliated with universities or research centers located in the southeastern region of Brazil. Similarly, the sample evaluated was predominantly (50%) from the Southeast region [16, 17, 19–23, 25, 30, 34, 36, 37, 39–41], followed by the South (6.67%) [16, 35], Northeast (6.67%) [18, 31], and Midwest (3.34%) [28]; 10 articles did not report the origin of the sample and none cited the North region as the origin of the sample (Fig. 2). The term “region of the country” refers to the geographical location of the Brazilian institutions responsible for the studies, categorized according to the second division of Brazil’s political map, as defined by the Brazilian Institute of Geography and Statistics (IBGE) [44]. Regarding study design, 23 of the 30 included studies did not report their methodological design. Among those that did report it, the most common designation was cross-sectional [18, 23, 24, 42].

**Table 2:** Included studies, authors, year of publication, and facial region analyzed

AUTHOR	YEAR OF PUBLICATION	SKULL/FACE ACQUISITION	FACE/REGION
Baccarin, et al. [14]	2024	CBCT	all face - FSTT
Baldasso, et al. [15]	2020	Photogrammetry	all face - FSTT
Barros, et al. [16]	2021	CBCT	all face -FSTT
Beaini, et al. [17]	2021	CBCT	all face -FSTT
Da-Silva, et al. [18]	2018	CT	all face -FSTT
De Almeida, et al. [19]	2013	cadavers - Needle and calipers	all face -FSTT
Fernandes, et al. [20]	2012	CT	all face -FSTT; Hair; Eyelashes; Eyebrows
Fernandes, et al. [21]	2013	CT	all face -FSTT; Hair; Eyelashes; Eyebrows
Fernandes, et al. [22]	2015	CT	all face -FSTT; Hair; Eyelashes; Eyebrows
Gomes, et al. [23]	2020	CBCT	all face -FSTT
Herrera, et al. [24]	2016	CT	all face -FSTT
Kuhnen, et al. [25]	2021	CBCT	all face -FSTT
Miranda, et al. [26]	2018	CT	all face - FSTT
Moraes, et al. [27]	2022	CBCT	all face - FSTT
Moritsugui, et al. [28]	2022	CBCT	all face -FSTT
Pereira, et al. [29]	2017	photogrammetry	all face -FSTT
Pereira, et al. [30]	2021	Photogrammetry	all face -FSTT
Pithon, et al. [31]	2014	Digital lateral teleradiographs	all face -FSTT
Rocha, et al. [32]	2003	Spiral CT	all face -FSTT
Romeiro, et al. [33]	2014	CT	all face -FSTT
Santos, et al. [34]	2008	MRI	all face -FSTT
Silvestre, et al. [35]	2024	CBCT	all face -FSTT
Tedeschi-Oliveira, et al. [36]	2009	Cadavers - Needle and calipers	all face -FSTT
Gomes, et al. [37]	2021	CBCT	Nose
Strapasson, et al. [38]	2017	CBCT	Nose
Strapasson, et al. [39]	2019	CBCT	Nose
Strapasson, et al. [40]	2020	CBCT	Nose
Tedeschi-Oliveira, et al. [41]	2016	Cephalometric radiographs	Nose
Dias, et al. [42]	2016	CBCT	Mouth
Fugiwara, et al. [43]	2024	Photo	Ear

Legend Cone-beam computed tomography (*CBCT*), facial soft tissue thickness (*FSTT*), computed tomography (*CT*)

Sample sizes ranged from 1 to 600 individuals [15, 20–22, 27, 29, 41], including both living individuals and cranial models, totaling 3,239 evaluated samples. Approximately half of the studies [14–16, 20–24, 27, 28, 31, 35, 37, 39, 40, 42, 43] reported samples composed predominantly of female individuals, although some studies did not specify the sex distribution. The reported age range varied from 6 to 90 years [14, 36], with a predominance of adult populations. With regard to the geographic origin of the samples, only a limited number of studies specified the exact region of Brazil, which may affect the reproducibility and broader population applicability of the proposed methodologies.

Craniofacial structures were predominantly obtained using cone-beam computed tomography (CBCT) and CT. Regarding the vital status of the participants, the vast majority of the investigations were conducted on living individuals, with only two studies (6.67%) [19, 36] evaluating cadaveric samples using needles and calipers. Concerning image acquisition positioning and its effect on tissue displacement (erect vs. supine), 13 studies based on CBCT [14, 16, 17, 23, 25, 27, 28, 35, 37–40, 42], 4 on photogrammetry and photo [15, 29, 30, 43], and 2 on conventional/cephalometric radiographs [31, 41] collected data with subjects in an erect (orthostatic) position. Conversely, the eight studies employing conventional medical CT [18, 20–22, 24, 26, 32, 33] and the single study based on Magnetic Resonance Imaging (MRI) [34] evaluated participants in the supine (dorsal decubitus) position.

**Fig. 3:**
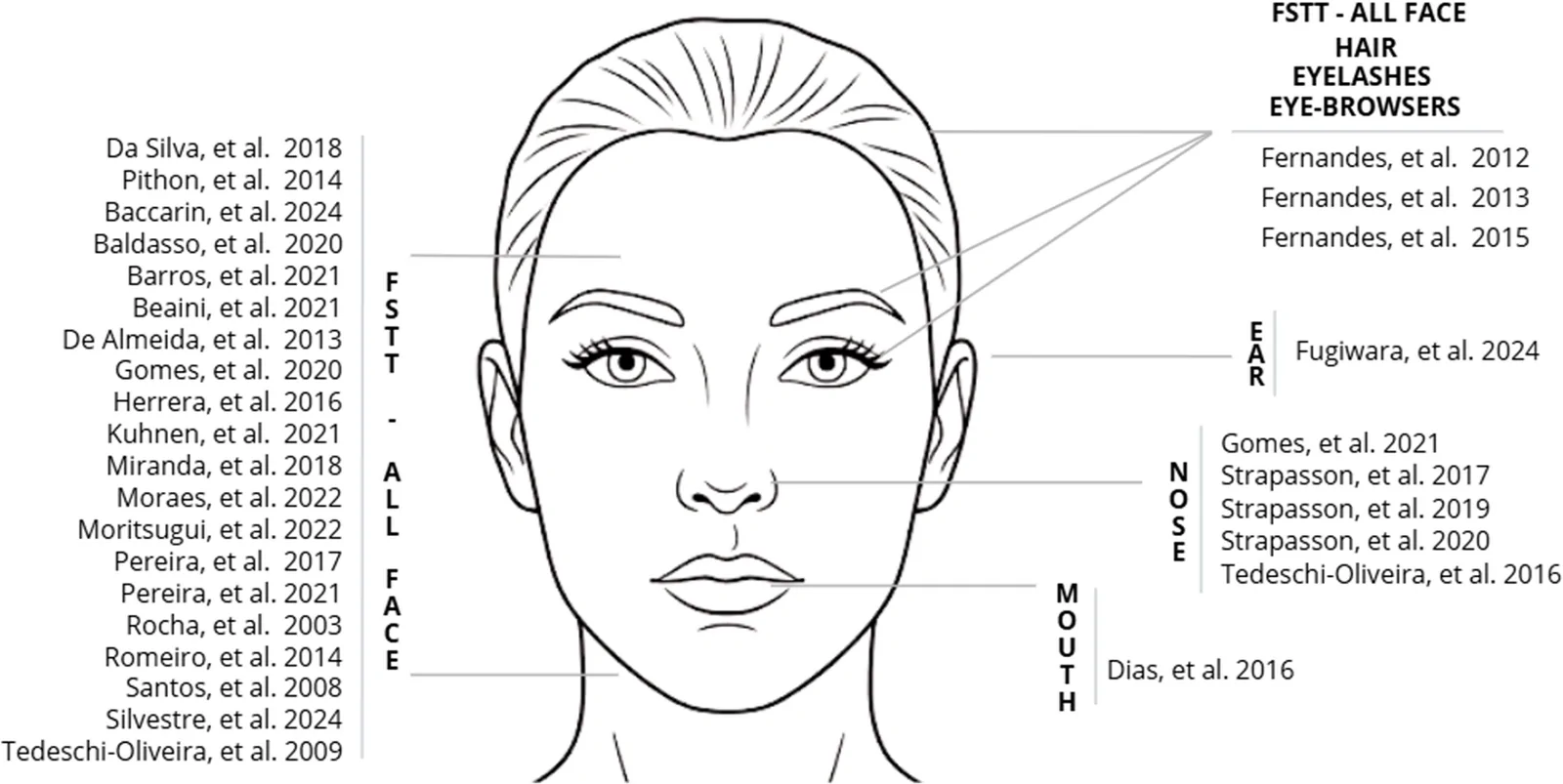
Distribution of the included studies according to the facial region investigated (nose, mouth, ear, and facial soft tissue thickness), indicating authors and year of publication. Legend FSTT = facial soft tissue thickness

Most studies performed complete facial approximation or evaluated FSTT [14–36], whereas others focused on specific facial regions, including the nose [37–41], mouth [42], ear [43], and accessory structures such as hair, eyebrows, and eyelashes [20–22] (Fig. 3).

Among the 23 studies that evaluated FSTT or simulated the entire face [14–36], 6 compared different age groups [14, 16–18, 25, 35], 9 assessed sex-related differences [16–19, 23, 25, 35, 36], 4 analyzed skeletal class [17, 23, 31, 35], and 2 evaluated nutritional status [18, 36] and differences between White and Black individuals [16, 36]. In six studies, comparisons were conducted between different methodologies or reference tables for FSTT [20–22, 24, 27, 29]. Additionally, two studies [14, 34] aimed to identify and describe the craniometric landmarks relevant for facial approximation.

### FFA development and operators

**Fig. 4:**
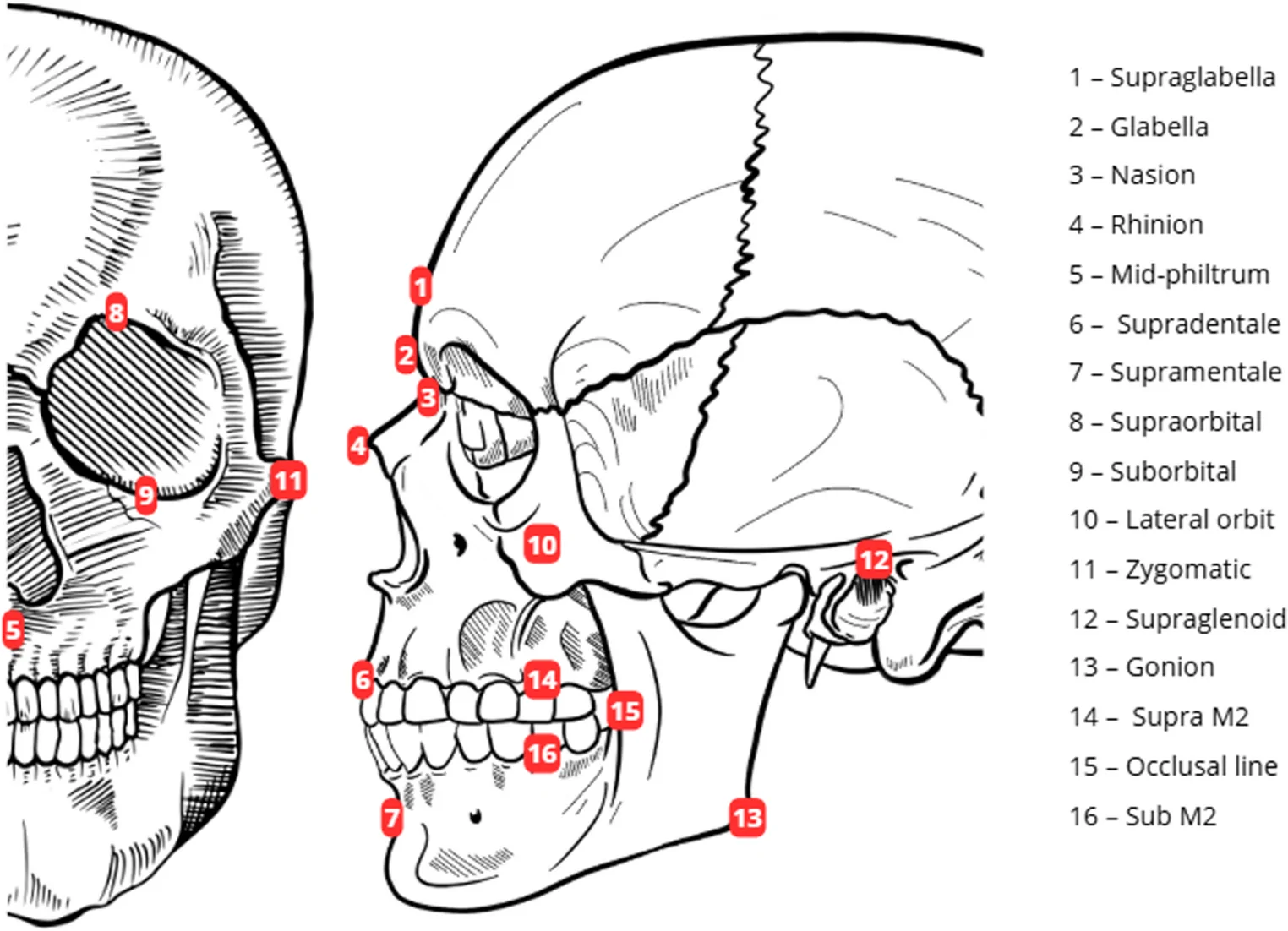
Representation of the anatomical landmarks most frequently described in the literature for forensic facial approximation. Legend: M2 (second molar)

The studies analyzed employed a variable number of anatomical landmarks, ranging from 3 to 44 points. Studies assessing FSTT used between 8 and 57 landmarks, while nasal approximation studies used between 3 and 6 points. The study focusing on mouth approximation used 13 landmarks, whereas the study addressing ear approximation used 9 points. Of the points mentioned, 16 were repeated in more than 50% of the studies evaluated, while 47 appeared in five or fewer works (Online Resource 3). The definitions and nomenclature of anatomical landmarks were relatively consistent across the studies, enabling identification of the most frequently reported landmarks (Fig. 4). A total of ten different software programs were used for craniofacial approximation and measurement, including both commercial and open-source tools, with Blender and OsiriX being the most frequently used.

The number of operators involved in the landmarking or reconstruction process ranged from one to three; however, most studies did not report whether the operators had prior experience or specific training [14–23, 25–27, 29, 31, 32, 34, 36]. Only 14 studies reported performing reliability tests to assess intra- or inter-examiner agreement [14, 16, 17, 19, 23, 25, 28, 31, 32, 37, 39–41, 43], with the Intraclass Correlation Coefficient (ICC) being the most frequently reported measure. In eight studies [15, 20–22, 24, 26, 27, 30], the objective was to evaluate simulated FFA. The number of evaluators ranged from 1 to 120 [24, 27]; however, one study did not report this information [15], and another employed software to perform this evaluation [26].

Overall, the studies mapped in this scoping review reported results primarily based on measurements of FSTT, with data commonly expressed as means and standard deviations. The analyses included different population groups, encompassing children, subadults, and adults of both sexes, with stratifications according to age, sex, nutritional status, facial typology, skeletal class, and ethnic classification. Only one study [28] reported a comparison with another geographic region of the country, finding a predominance of deeper FSTT in the Central-West sample when compared to the Southeast sample.

Results related to FRR included recognition and similarity rates of the target individual, expressed as percentages of correct identification. These outcomes varied according to the FSTT standards employed, the approximation technique used, and the group of evaluators involved [20–22, 24, 26, 27, 30]. Some studies [20–22, 24] also reported the absolute numbers and percentages of examiners who correctly recognized the target individual under different approximation protocols.

Additional findings included the development of statistical models describing relationships between hard and soft tissue measurements, as well as equations proposed to estimate facial dimensions such as nasal width and area [39], nasal projection [41], vermilion height [42], mouth width [42], and correspondences between cranial measurements and ear morphology [43].

The analyzed studies also reported the presence of sexual dimorphism in FSTT, with values generally higher in males than in females [16, 17, 25, 28, 35, 36, 38, 42, 43]. This pattern was particularly observed at craniofacial midline landmarks. Several authors [16, 17, 25, 28, 36, 42] have suggested that the main sex-related differences are concentrated in the lower facial region; however, no clear consensus exists regarding this finding or the specific landmarks exhibiting sexual dimorphism.

Similarly, there is no agreement concerning the number of affected landmarks or the specific facial regions in which females may present higher values. In some lateral facial regions, higher mean FSTT values were reported in females, contrasting with the overall male-dominant pattern [18, 28].

Regarding age, most mapped studies [16, 18, 25] indicate that it influences FSTT, with a tendency toward higher values in adults and older individuals compared with children and young adults. Despite this trend, the studies [16, 18, 25] suggest that the effect of age on FSTT is not homogeneous across the face, varying according to the anatomical region analyzed, as well as individual and methodological characteristics. Similarly, the studies consistently report that skeletal class and facial type exert a significant influence on FSTT [17, 23, 35] and on nasal positioning [37]. Despite the overall influence of skeletal class and facial type, one study [31] reported differences restricted to specific anatomical landmarks, without generalized variations across all evaluated classes.

Another factor identified was nutritional status, expressed by body mass index (BMI). The analyzed studies [18, 36] indicate that this variable significantly influences FSTT. Individuals with BMI above the normal range showed higher FSTT values across a broad set of anatomical landmarks, including both midline and bilateral regions. No consensus was observed regarding the influence of skin color on FSTT among the evaluated studies [16, 36]. All studies reported proposing a method with potential forensic applicability or developing Brazilian reference tables. However, not all articles reported funding sources or declared conflicts of interest, which limits methodological transparency.

## Discussion

The present study aimed to map FFA methodologies applied to the Brazilian population, encompassing approaches ranging from FSTT measurements to techniques aimed at reconstructing specific facial structures. The results, synthesized from 30 studies, indicate the growing investigation of this auxiliary identification tool within a high population variation. This recent increase, particularly evident after 2015, reflects the expansion of access to imaging technologies and the broader availability of specialized software, factors that Gupta et al. [1] highlight as milestones in the modernization of FFA. However, this technological advancement has not been accompanied by a proportional level of methodological standardization among national studies.

A notable methodological concern identified in this review was the absence of a clearly reported study design in the majority of the mapped studies. This tendency may be related to the interdisciplinary nature of FFA, in which the focus on technical validation and the adaptation of tools often precedes the formalization of methodological design in scientific reports, as observed in investigations aimed at standardizing anatomical landmarks using emerging technologies [34]. This gap compromises transparency and reproducibility in light of reporting guidelines such as PRISMA-ScR and STROBE. Furthermore, the high concentration of studies in the Southeast region of Brazil (83.3%) may be associated with the greater density of postgraduate programs and forensic research centers in this area. As discussed by Nunes et al. [45], this geographical bias is particularly critical in a country with extensive morphological diversity, since there is an ancestry gradient associated with regional population variation, which limits the national applicability of the proposed FSTT standards and can lead to errors when applied in different forensic contexts.

The variation between orthostatic and supine positioning among the mapped acquisition methods represents an important source of methodological heterogeneity in the standardization of FSTT tables in Brazil. In CBCT and photos/photogrammetry studies, participants are generally positioned upright [14–17, 23, 25, 27–31, 35, 37–43], reproducing the natural action of gravity on facial soft tissues. In contrast, medical CT and MRI examinations are commonly performed with individuals in the supine position [18, 20–22, 24, 26, 32–34], which may alter soft tissue distribution due to gravitational effects. Additionally, the cadaveric studies included in this review [19, 36] may also be influenced by post-mortem changes such as dehydration and loss of muscle tone. Together, these factors may limit the direct comparability between published reference tables and contribute to variability when population-specific parameters are extrapolated across different FFA protocols.

Although several Brazilian studies identified statistically significant differences in FSTT according to sex, age, skeletal class, and nutritional status, the practical forensic impact of these variations remains debated. Previous studies have suggested that some population-specific differences in soft tissue depth may be relatively small and concentrated in less projecting facial regions, mainly influencing facial contours rather than highly recognizable structures such as the eyes, nose, and mouth [1, 43]. Consequently, statistically different FSTT values do not necessarily produce perceptible differences in facial recognition outcomes. In fact, the forensic literature has long established that FFA is not intended to produce an exact replica of the individual, but rather an anatomical approximation capable of assisting recognition processes [1, 6, 24, 43]. Nevertheless, even if some differences are subtle when individually analyzed, minimizing anatomical discrepancies through population-specific parameters may contribute to greater anatomical plausibility and facial fidelity, particularly when multiple small variations are cumulatively incorporated during reconstruction. Therefore, despite the importance of developing Brazilian reference standards, future studies should also investigate the actual visual and recognition impact of population-specific FSTT variations under real forensic conditions.

In addition to the lack of clear reports on study designs, critical analysis of the results reveals weaknesses in the validation of the human factor in Brazilian research on FFA. The fact that only half of the studies reported intra- and inter-examiner agreement tests, along with the absence of information on operator training and prior experience in more than a quarter of the studies, suggests that the reported accuracy may be subject to uncontrolled errors in the identification of reference points. Differences in operator experience can influence reconstruction results and reduce reproducibility between studies. Consequently, the lack of standardization may compromise the reliability and future validation of national protocols, reinforcing the need for future Brazilian studies to adopt standardized guidelines for report preparation, such as the recommendations of study design guidelines.

In this context, the results reveal a paradox regarding influencing factors: while sexual dimorphism appears fragmented and, at times, contradictory with females exceeding male averages at specific lateral landmarks [24, 34], BMI and skeletal class demonstrate a global and consistent influence across most facial landmarks. This finding suggests that FFA studies in Brazil should move beyond purely binary reference tables toward multifactorial models that prioritize nutritional status as a more robust morphological predictor than sex alone.

From a methodological perspective, a predominance of studies focusing on FSTT across the entire face was observed, indicating greater practical adherence to the American and British methods at the expense of the Russian anatomical method. This preference may be explained by lower technical complexity and reduced reliance on artistic skills. However, when compared with the current state of the art, Omstead [46] and Gupta et al. [1] warn that exclusive reliance on mean tissue-depth values may limit anatomical fidelity, particularly in structures with limited direct skeletal support, such as the nose, mouth, and ears. The scarcity of national studies focusing on these structures reinforces the urgent need to standardize parameters that extend beyond thickness tables, aiming to reduce empiricism in the simulation of these highly subjective anatomical regions.

Scientific transparency, therefore, emerges as a fundamental pillar for the transition from academic research to forensic practice. The absence of declarations regarding funding sources and conflicts of interest in part of the mapped sample represents an obstacle to contemporary standards of scientific integrity. For national reference standards to be incorporated into official forensic protocols, clarity regarding potential biases and institutional support behind the investigations is as essential as the statistical rigor of the reported data, ensuring the admissibility of these findings in legal contexts.

Finally, the efforts of Santos [34], who described the localization of anatomical landmarks using MRI, and Baccarin [14], who adapted these landmarks to the complexity of pediatric populations, reinforce that the accuracy of FFA extends beyond computational capacity alone. These studies demonstrate that the correct identification and standardization of anatomical landmarks constitute the fundamental pillars determining the reliability of the technique.

In summary, although national scientific production has advanced in adapting international methodologies, the strong concentration on a single methodological axis and the heterogeneity of protocols still hinder the consolidation of universal guidelines, preventing FFA from becoming a fully robust and validated tool within the Brazilian forensic context.

## Conclusion

In conclusion, Brazilian scientific production on FFA has advanced considerably in recent years, particularly regarding the establishment of FSTT references and the adaptation of international methodologies for populations with high heterogeneity. However, important methodological limitations persist, including heterogeneous protocols, incomplete reporting standards, and regional concentration of studies. Although population-specific differences in FSTT may not always result in perceptible differences in recognition outcomes, the use of anatomically and demographically appropriate parameters may contribute to greater facial plausibility during reconstruction procedures. Since FFA is inherently an auxiliary and approximative method rather than an exact facial reproduction, future research should focus not only on expanding representative Brazilian databases but also on investigating the real visual and forensic impact of population-specific variations under practical identification conditions.

## Supplementary Information

Below is the link to the electronic supplementary material.

Supplementary Material 1 (PDF 476.2 KB)

Supplementary Material 2 (PDF 75.4 KB)

Supplementary Material 3 (PDF 76.2 KB)

## Data Availability

Since this is a scoping review, all data supporting the conclusions of this study are available in the article and its Supplementary Information.
