# Supplementary material for: Forensic facial approximation methodologies applied to the Brazilian population: a scoping review
Source: Int J Legal Med. 2026 Aug 8;140(6):3611–21. doi: 10.1007/s00414-026-03947-2 (PMC13658438; doi:10.1007/s00414-026-03947-2)
Supplement: Supplementary file 1 [file 414_2026_3947_MOESM1_ESM.pdf]

Forensic Facial Approximation Methodologies Applied to the Brazilian Population: scoping review

International Journal of Legal Medicine

Paula de Sousa Rachid[1], Daniel Pignatari Mahet Rodrigues[1], Lucas Alves Jural[2], Andrea Vaz Braga Pintor[3], Lucianne Cople Maia[4], Maria Augusta Visconti[4], Andreia Cristina Breda de Souza[4]

[1]DDS, MSc – Faculty of Dentistry of the Universidade Federal do Rio de Janeiro. Rio de Janeiro/RJ – Brazil.

[2]DDS, PhD – Faculty of Dentistry of the Universidade Federal de Uberlândia. Minas Gerais/MG - Brazil.

[3]DDS, MSc, PhD. Faculty of Dentistry of the Universidade Federal Fluminense, Rio de Janeiro, RJ, Brazil.

[4]DDS, MSc, PhD – Faculty of Dentistry of the Universidade Federal do Rio de Janeiro. Rio de Janeiro/RJ – Brazil.

Corresponding author: andreiacrisbreda@gmail.com

Online Resource 1.

Data extracted separately by study, presenting general information and details of the population and sample used.

GENERAL INFORMATION			POPULATION			
			Sample			
Author/Year/ Origin Of The Main Author	Study Design	Objective	Total	Sex	Age	Geographic Region Of The Country
Baccarin, 2024/ SP	NR	Report the method for measuring the average FSTT of 32 landmarks adapting the method for adults replacing the landmarks at the upper and lower second molars (Supra M2 and Infra M2) in children up to 10 years of age for a measurement using the deciduous second molars as reference.	47	24F 23M	06-10y	NR
Baldasso, 2020/ RS	case report	To report on the successful implementation of computer-aided forensic facial approximation in a real case in the forensic context of the General-Institute of Forensic Expertise, RS, Brazil.	1	1M	between 23 and 30 years old	South
Barros et al, 2021/ SP	NR	Measure FSTTs of Brazilian adult individuals, based on CBCT images, considering the variables sex,age, ancestry and nutritional status.	106	71F 35M	19-32y and +32y	Southeast
Beaini, 2021/ MG	NR	Is to detailly describe a method for soft tissue thickness measurement in CBCT DICOM files and to test it in the analysis of a multi-ancestral Brazilian sample, subdivided into sex, age and FGP.	100	50 F 50M	18-65Y	Southeast
Da Silva, 2018 / PE*	cross section	Create a database for determining soft tissue thickness and initiate this pilot study, aiming at future three-dimensional facial reconstructions of Brazilians to apply in recognition of bone remains, as	101	39F 39M	39,30 ± 17,61	North East

		well as compare these results with other populations around the world.			(36,00)	
De Almeida, 2013/ SP	NR	Tests traditional and novel landmarks of facial tissue thickness in adult cadavers from Brazil.	100	26F 74M	41-60y	Southeast
Fernandes, 2012/ SP	NR	Focuses on the performance of three different characterized digital forensic facial reconstructions (using hair, eyelashes and eyebrows) of a Brazilian subject (based on an international database and two Brazilian databases for soft facial tissue thickness), and evaluates the digital forensic facial reconstructions comparing them to photos of the target individual and nine other subjects.	1	1F	Between 33 and 47y	Southeast
Fernandes, 2013/ SP	NR	This study focuses on the performance of three different digital forensic facial reconstructions, without hair, of a Brazilian female subject (based on one international database and two Brazilian databases for soft facial-tissue thickness). It evaluates the digital forensic facial reconstructions comparing them to photographs of the target individual and nine other subjects, employing the recognition method.	1	1F	Between 33 and 47y	Southeast
Fernandes, 2015/SP *	NR	This work aimed to analyze 3D digital forensic facial reconstructions and, based on scientific evidence, propose a pilot protocol for their execution and presentation.	1	1F	Between 33 and 47y	Southeast
Gomes, 2020 / SP	cross section	Evaluate the influence of skeletal class, facial type, and sex on soft tissue thickness in the craniofacial midline to provide data for reliable facial reconstructions in the Brazilian population.	121	67F 54M	21-40y	Southeast
Herrera, 2016 / SP	NR	Investigate the performance of four FSTT datasets in the recognition and resemblance of individuals. It also sought to analyze the performance of assessors in recognizing people, according to sex and knowledge on Human Anatomy and Forensic Dentistry.	4	2-F 2M	NR	NR
Kuhnen, 2021 / SP	NR	Measure the thickness of soft tissues of Brazilian subadults, based on data from Cone-Beam Computed Tomography (CT) images of living individuals, considering the variables sex, age, ancestry and body mass index (BMI)	126	66F 60H	7-18y	Southeast
Miranda, 2018 / SP	NR	Evaluate the accuracy and recognition level of 3D CCFR using free open-source software programs.	4	2F 2M	21-49y	NR
Moraes, 2022 / RS	NR	Present two protocols of Two-Dimensional Facial Approximation, ultimately, to subsidize part of the recognition of unidentified individuals.	1	F	between 30-40y	NR
Moritsugui, 2022 / SP	NR	Measure the FSTT in a Brazilian sample from the Midwest region and compare to a previous sample from the Southeast region using the same cone-beam computed tomography (CBCT) measuring protocol.	101	56F 45M	18-41y +42y	Midwest region
Pereira, 2017 / SP *	NR	Perform RFF on the same artificial skull used for educational purposes using both digital and manual methods, and compare the results obtained, as well as the difficulties and differences encountered between the two techniques.	1	M	between 30-40y	NR

Pereira, 2021 / SP	NR	Evaluate a capacity of unfamiliar face matching by facial approximation in skulls whose faces are known, and to verify the existence of an association between level of understanding of the subject by the evaluators and the recognitions matches.	16	NR	NR	Southeast
Pithon, 2014 / BA	experimental approach	Evaluate the variation in facial soft tissue thickness in young north eastern Brazilian individuals according to gender and skeletal class.	300	156F 144M	8-12y	North eastern
Rocha, 2003 / SP	NR	To present an innovative methodology for forensic individual identification, by determining anthropometric measurements using 3D-CT computergraphics resources.	5	NR	NR	NR
Romeiro, 2014 / RJ	NR	To produce automatic facial reconstructions with all the soft tissue structures without being biased toward predefined templates.	3	1F 2M	NR	NR
Santos, 2008 / SP *	NR	Locate and define, in MRI scans, 22 craniometric points to be used in facial reconstructions for forensic purposes, in order to allow the future validation of a method involving the measurement of soft tissues of the faces of living individuals, which will enable the creation of a table of thickness measurements of soft tissues of the faces of living Brazilians.	NR	M	NR	Southeast
Silvestre, 2024 / PR	observational, longitudinal, and retrospective	Analyze the influence of skeletal class, occlusal class, age, and sex on the thickness of facial soft tissues in a sample of the Brazilian population.	239	139F 100M	18-78y	South
Tedeschi-Oliveira, 2009 / SP	NR	Assess the measurements of the thickness of soft tissue that covers anatomical landmarks of the skull using a population sample of corpses at the "Seção Técnica de Verificação de Óbitos" in Guarulhos, São Paulo.	40	14 F 26 M	17-90y	Southeast
Gomes, 2021 / SP	cross-sectional	Evaluate the influence of sex, skeletal class (class I, II and III), and facial type (brachycephalic, mesocephalic and dolichocephalic) on the nasal dimensions of Brazilian individuals, using CBCT images.	121	67F 54M	21-40y	Southeast
Strapasson, 2017 / SP	NR	Evaluate the relationship between the alar cartilage and piriform aperture using cone beam computed tomography (CT) imaging.	96	47 F 49 M	18-65y	NR
Strapasson, 2019 / SP	NR	Determine parameters to estimate the nasal width in forensic facial approximation, to analyze if there is a correlation between nasal profile and facial type, and to test the linear regression equations proposed to estimate nasal width in Brazilian subjects.	246	183 F 63 M	18-65y +65y	Southeast
Strapasson, 2020 / SP	NR	Validate the method presented by Tedeschi-Oliveira et al.24 using a different Brazilian sample and CBCT exams.	228	171 F 57 M	24-86y	Southeast
Tedeschi-Oliveira, 2016 / SP	NR	Determine the nasal projection point (Pn).	600	300 F 300 M	24-77y	Southeast

Dias, 2016 / SP	cross section	To construct a pattern of reference for the dimensions and proportions of the lips and to estimate the height and width of the mouth by means of hard-tissue measurements in adults.	430	sample 1: 185F 137M sample 2: 68F 40M	NR	NR
Fugiwara, 2024 / SP	NR	Investigated the size, position, and inclination of the ears and their possible relationships with adjacent tissues by analysing pre-established indices and new hypotheses to improve forensic facial reconstruction	99	55F 44M	18-65y	NR

Legend: F- female; M-male; y- years; NA- Not applicable; NR - Not reported; * Article in Portuguese, translated by the author.

Data extracted separately by study and information on the methodology used.

**Author/ Year**	**Method**									
	**Skull/Face Acquisition**	**Face/ Region**	**Anatomic Landmarks**		**Software**		**Evaluators/Operators**			
			**N°**	**Landmarks**	**Skull/Face Reconstruction**	**Reconstruction/ Measurement**	**Operators Of Reconstruction**	**Previous Training/ Experience**	**Kappa/ Icc**	**Evaluator**
Baccarin et al, 2024	CBCT	all face - FSTT	21 Total: 32	Supraglabella, Glabella, Nasion, Rhinion, Mid-philtrum, Labrale Superius, Labrale Inferius, Supramentale, Pogonion, Menton, Frontal Eminence, Mid-supraorbital, Mid-infraorbital, Malar, Lateral Orbital, Zygion, Supraglenoid, Gonion, Deciduous Supra M2, Occlusal Line, Deciduous Infra M2	HOROS	HOROS	2	NR	ICC Intra - 0,98 Inter - 0,93	NA
Baldasso, 2020	Photogrammetry	all face - FSTT	21	NR	Autodesk 123D	Blender	NR	NR	NR	NR

Barros et al, 2021	CBCT	all face -FSTT	21 Total: 32	Supraglabella, Glabella, Nasion, Rhinion, Mid-philtrum, Supradentale, Infradentale, Supramentale, Mental eminence, Menton, Frontal Eminence, Supraorbital, Suborbital, Inferior malar, Lateral orbit, Zygomatic, Supraglenoid, Gonion, Supra M2, Occlusal line, Sub M2 Point	Osirix Lite	Osirix Lite	1	NR	TEM=0,56 rTEM=4,90%	NA
Beaini, 2021	CBCT	all face -FSTT	21 Total: 32	Infraorbital, Porion, Basion, Supraglabella, Nasion, Rhinion, Mid-Philtrum, Labrale Inferius, Supramentale, Pogonion, Gnathion, Frontal Eminence, Supraorbitale, Suborbitale, Malar, Lateral Orbital, Zygion, Gonion, Supraglenoid, Supra M2, Occlusal Line, Sub M2	Osirix Lite	Osirix Lite	2	NR	ICC Reproducibility was high in linear and intraclass correlation (>7) in all intraobserver measurements and in 26/32 points of the interobserver measurements.	NA
Da Silva, 2018*	TC	all face -FSTT	20	Supraglabella, Glabella, Nasion, Rhinion, Mid-philtrum, Supradentale, Infradentale, Supramentale, Pogonio Menton, Supraorbital, Infraorbital, Inferior malar, Lateral orbit, Zygomatic, Supraglenoid, Gonion, Supra M2, Occlusal line, Sub M2 Point	InVesalius 3,0	InVesalius 3,0	NR	NR	NR	NA

De Almeida, 2013	cadavers - Needle and callipers	all face -FSTT	31	Supraglabella, Glabella, Nasion, Rhinion, Supratip break, Mid-philtrum, Upper lip margin, Superior labial sulcus, Stomion, Lower lip margin, Chin-lip fold, Pogonion, Beneath chin, Frontal eminence, Supraorbital, Suborbital, Ecthocanthion, Endocanthion, Inferior malar, Lateral orbit, Zygomatic arch, Supraglenoid, Alare, Gonion, Supra-M2, Occlusal line, Infra-M2, Supra-canine, Infra-canine, Cheilion, Mid-ramus	corpse	NA	1	NR	ICC (0,604 - 0,969)	NA
Fernandes, 2012	TC	all face -FSTT Hair, Eyelashes, Eyebrows	21 Total: 32	Supraglabella, Glabella, Nasion, Rhinion, Mid-philtrum, Supradentale, Infradentale, Supramentale, Mental eminence, Menton, Frontal eminence, Supraorbital, Suborbital, Inferior malar, Lateral orbit, Zygomatic arch, Supraglenoid, Gonion, Supra M2, Occlusal line, Sub M2	Mimics 1	3D Studio Software Max1	1	NR	NR	30
Fernandes, 2013	TC	all face -FSTT Hair, Eyelashes, Eyebrows	21 Total: 32	Supraglabella, Glabella, Nasion, Rhinion, Mid-philtrum, Supradentale, Infradentale, Supramentale, Mental eminence, Menton, Frontal eminence, Supraorbital, Suborbital, Inferior malar, Lateral orbit, Zygomatic arch, Supraglenoid, Gonion, Supra M2, Occlusal line, Sub M2	Mimics 1	3D Studio Software Max1	1	NR	NR	22
Fernandes, 2015*	TC	all face -FSTT Hair, Eyelashes, Eyebrows	NA	NR	Mimics	3DS MAX	NR	NR	NR	22

Gomes, 2020	CBCT	all face -FSTT	20	Glabella, Glabella', Nasion, Nasion', Rhinion, Rhinion' Subspinale, Subnasal, Prosthion, Labrale superius', Incision, Stomion, Infradentale, Labrale inferius', Supramentale, Supramentale', Pogonion, Pogonion', Gnathion, Gnathion'	NemoCeph	NemoCeph	2 Radio<br>1 Ortho	NR	TEM, rTEM and R indicated high reliability, with most intra-examiner rTEM <5%, TEM <1 mm, and >90% of variation due to external causes.	NA
Herrera, 2016	TC	all face -FSTT	NA	NA	InVesalius Magics	Manual	1	No	NR	120
Kuhnen, 2021	CBCT	all face -FSTT	21 Total: 32	Supraglabella, Glabella, Nasion, Rhinion, Mid-Philtrum, Supradentale, Infradentale, Supramentale, Mental eminence, Menton, Frontal eminence, Supraorbital, Suborbital, Inferior malar, Lateral orbit, Zygomatic arch, Supraglenoid, Gonion, Supra M2, Occlusal line, Sub M2	OsiriX Lite	OsiriX Lite	NR	NR	TEM and rTEM<br><br>Mean TEM was 0.64 mm (-0,25;-1,17mm), and mean rTEM was 6.83% (-3,63%;13,44%).	NA
Miranda, 2018	TC	all face - FSTT	21 Total: 32	Supraglabella, Glabella, Nasion, Rhinion, Mid-Philtrum, Supradentale, Infradentale, Supramentale, Mental eminence, Menton, Frontal eminence, Supraorbital, Suborbital, Inferior malar, Lateral orbit, Zygomatic arch, Supraglenoid, Gonion, Supra M2, Occlusal line, Sub M2	Horos1 (version 3.0)	Blender1 2.78 version	1	NR	NR	CloudCompare1 2.6.3 software
Moraes, 2022	CBCT	all face - FSTT	21 Total: 32	Supraglabella, Glabella, Nasion, Rhinion, Mid-Philtrum, Supradentale, Infradentale, Supramentale, Mental eminence, Menton, Frontal eminence, Supraorbital, Suborbital, Inferior malar, Lateral orbit, Zygomatic arch, Supraglenoid, Gonion, Supra M2,	Blender (version 2.75a -OrtogonBlender5)	Adobe Photoshop 2021/Blender version 2.75a -OrtogonBlender5/ MakeHuman (version 1.0.2)	2	NR	NR	1

				Occlusal line, Sub M2						
Moritsugui, 2022	CBCT	all face -FSTT	21 Total: 32	Supraglabella, Glabella, Nasion, Rhinion, Mid-Philtrum, Prosthion, Infradentale, Supramentale, Pogonion, Menton, Frontal Eminence, Mid-Supraorbital, Mid-infraorbital, Inferior malar, Lateral orbit, Zygomatic arch, Supraglenoid, Gonion, Supra M2, Occlusal line, Sub M2	Horos version 3.3.6	NA	2	Yes	ICC intra- and interexaminer agreement above 95%	NA
Pereira, 2017*	photogrammetry	all face -FSTT	21 Total: 32	Supraglabella, Glabella, Nasion, Rhinion, Mid-Philtrum, Prosthion, Infradentale, Supramentale, Pogonion, Menton, Frontal Eminence, Mid-Supraorbital, Mid-Suborbital, Inferior malar, Lateral orbit, Zygomatic arch, Supraglenoid, Gonion, Supra M2, Occlusal line, Sub M2	Recap 360º	Blender® and Manual	1	NR	NR	NA
Pereira, 2021	Photogrammetry	all face -FSTT	21	Supraglabella, Glabella, Nasion, End of Nasal, Mid-Philtrum, Upper lip margin, Lower lip margin, Chin-lip fold, Mental eminence, Beneath chin, Frontal Eminence, Supraorbital, Suborbital, Inferior malar, Lateral orbit, Zygomatic arch, Supraglenoid, Gonion, Supra M2, Occlusal line, Sub M2	Autodesk Recap Studio	Blender®	1	Yes	NR	20
Pithon, 2014	Digital lateral teleradiographs	all face -FSTT	10	Glabella, Nasion, Rhinion, Subnasal, Upper Lip, Stomion, Bottom lip, Labiomental region, Pogonion, Gnathion	Sidexis Xg program	NA	1	NR	Kappa= 0,79 Taken together, the data evaluated from to TEM and rTEM and R demonstrated an acceptable accuracy in this study.	NA

Rocha, 2003	Spiral CT	all face -FSTT	8	Nasal opening, Glabella, Menton, Nasio, Nasiospinale, Opisthocranium, Porion; Zygion	Vitrea (version 2.3)	NA	2	NR	Standard error percentage of 1.95% (bone) and 1.61% (soft tissue).	NA
Romeiro, 2014	TC	all face -FSTT	44 Total: 57	Supraglabella, Glabella, Nasion, Rhinion, Prosthion / Supradentale, Infradentale Skin thickness, Supramentale, Gnathion / Mental eminence, Subgnathion / Menton, Frontal eminence, Supraorbital, Suborbital, Inferior malar , Lateral orbit, Zygomatic arch , Supraglenoid, Gonion , Supra M2, Occlusal line , Sub M2, Lateral glabella, Lateral nasal, Mid lateral orbit, Mid masseter , Supra canina, Sub canina, Mental tubercule anterior, Mid mandibular, Porion, Bregma, Acanthion / Nasospinale, Subnasal / Subspinale, Lateral piriform margin, Medial orbital margin, Supra orbital margin, Lateral orbital, Infra orbital margin, Posterior lateral orbital margin, Superior central incisor, Superior lateral incisor, Superior canine, Superior first premolar, Superior second premolar, Infraorbital foramen	NR	NR, however, utilizes Hermite Radial Basis Functions technology.	1	a professiona l in the field of forensic medicine	NA	NA

Santos, 2008*	MRI	all face -FSTT	22 Total: 33	Trichion, Supraglabella, Glabella, Nasion, Rhinion or end of the nasal bone, Philtrum, Margin of the upper lip, Margin of the lower lip, Supramental, Mental eminence, Gnathion, Frontal Eminence, Superior Orbital, Inferior Orbital, Inferior Zygomatic or Lower Malar, Lateral Orbit or Zygomatic-Orbital, Middle of the Zygomatic Arch, Supraglenoid, Gonion, Supra M2, Occlusal Line, Sub M2	E-film	NR	NR	NR	NR	NA
Silvestre, 2024	CBCT	all face -FSTT	10	Glabella, Nasion, Rhinion, Subspinale, Prosthion, Incision, Infradentale, Supramentale, Pogonion, Gnathion	InVesalius	InVesalius	2	Yes	NR	NA
Tedeschi-Oliveira, 2009	Cadavers - Needle and callipers	all face -FSTT	21 Total: 32	Supraglabella, Glabella, Nasion, Rhinion, Mid-Philtrum, Supradentale, Infradentale, Supramentale, Mental eminence, Menton, Frontal eminence, Supraorbital, Suborbital, Inferior malar, Lateral orbit, Zygomatic arch, Supraglenoid, Gonion, Supra M2, Occlusal line, Sub M2	corpse	NA	2	NR	NR	NA
Gomes, 2021	CBCT	Nose	6	Nasion, pronasale, subnasale, alare, nasal wings, alar base	NemoCeph	NemoCeph	2	NR	ICC - Intra 0,997	NA
Strapasson, 2017	CBCT	Nose	04 Total: 8	alare, subalare, curvature, base	OsiriX (version 5.9)	OsiriX (version 5.9)	2	NR	NR	NA
Strapasson, 2019	CBCT	Nose	3	Rhinion, Pronasale, Prosthion	Horos 1.1.7	Horos 1.1.7	NR	NR	ICC intra 0.97–0.99	NA
Strapasson, 2020	CBCT	Nose	3	Rhinion, Pronasale, Prosthion	Horos 1.1.7	Horos 1.1.7	1	NR	ICC intra 0.99	NA

Tedeschi-Oliveira, 2016	Cephalometric radiographs	Nose	4	Rhinion, Pronasale, Prosthion, Pogonion'	Image J version 1.43n/JAVA 1.6 0_10	Image J version 1.43n/JAVA 1.6 0_10	2	NR	rTEM for the male (0.44%) and the female (0.40%) was <1%, showing good concordance.	NA
Dias, 2016	CBCT	Mouth	13	Nasospinale, Philtrum medium, Supradentale, Infradentale, Supramentale, Gnathion, Foramen incisivus inferius, Foramen incisivus superius, Labiale superius, Labiale inferius, Canini, Mentale, Cheilion	Osirix	Osirix	1	Yes	NR	NA
Fugiwara, 2024	Photo	Ear	12	Superaurale, Subaurale, Otobasion superius, Posterotragion, Ecthocanthion, Alar curvature, Glabella, Subnasale, Sellion, Menton, Zygion, Otobasion inferius	Photoshop 2021	Photoshop 2021	1	Yes	ICC intra- and inter-examiner 0.90 and 0.95,	NA

Legend: NA-Not applicable; NR - Not reported; TC - computed tomography; CBCT - cone beam computed tomography; FSTT- Facial Soft Tissue Thickness; MRI - Magnetic Resonance Imaging; TEM- Technical Error Measurement; rTEM-technical error of measurement expressed in percentage; R- coefficient of reliability; * Article in Portuguese, translated by the author.

Data extracted separately by study and information on the results and conclusions reached.

**Author/ Year**	**Outcome**				
	**Main Results Of The Methodology**		**Conclusion**	**Conflict Of Interest**	**Financing**
	**General Results**	**Detailed Results**			
Baccarin et al, 2024	Mean FSTT values - children of both sexes (6–10 years old).<br><br>Average soft tissue thickness of “permanent” M2 points in adults (Beaini et al, 2021) and “estimated“ M2 and ”Deciduous“ M2 points in children (6–10 years).	The average thickness analysis showed that a difference of less than 1.5 mm was observed between male and female participants.<br><br>The results of measurements taken on children aged 6 to 10 years at the "estimated" and "Deciduous" M2 points show less thickness than in adults.	"A methodology developed for measuring FSTT in CBCT examinations of adults and found that it was applicable to most of the described landmarks. A necessary technical adaptation to obtain data in the regions of deciduous second molars from both arches was also successfully tested."	No	Academic Cooperation Program in Public Security and Forensic Sciences (CAPES)

Baldasso, 2020	The forensic experts involved in the investigation recognized one individual and ruled out the possibility of other alleged victims.	NA	"This case report aims to expose a 3D facial approximation method, with subsequent recognition in the civil identification database of the state of Rio Grande do Sul, Brazil, by the forensic experts involved in the investigation, having subsequently confirmed the victim's identification by the DNA method."	NR	NR
Barros et al, 2021	Means and standard deviation of facial soft tissue thickness measurements , in Brazilian living adults.<br>Means and standard deviation of facial soft tissue thickness , in Brazilian living adults, according to sex.<br>Means and standard deviation of facial soft tissue thickness, in Brazilian living adults, according to age groups (19 to 32y; above 32).<br>Means and standard deviation of facial soft tissue thickness, in Brazilian living adults with normal and above normal BMI.<br>Means and standard deviation of facial soft tissue thickness, in white and black Brazilian living adults.	Statistically significant differences between men and women were found at 14 anatomical reference points (supraglabella, glabella, nasion, rhinion, middle philtrum, supradentale, infradentale, supramentale, mentum, frontal eminence, supraorbital, inferior malar, gonion, and occlusal line); differences were found between the groups aged 19 to 32 years and over 32 years (in the supraglabella, glabella, supradentale, infradentale, frontal eminence, supraorbital, suborbital, lateral orbit, gonion, and subM2), with older individuals showing higher FSTT values, except at the supradentale point. Seven points showed differences in FSTT between whites and blacks, with higher values for blacks (supraglabella, middle philtrum, mentum, frontal eminence, supraorbital, and suborbital). Fourteen points showed statistically significant differences between individuals with normal and above-normal BMI, with higher values for the latter (supraglabella, glabella, nasion, rhinion, frontal eminence, supraorbital, suborbital, inferior malar, lateral orbit, zygomatic arch, gonion, supra M2, occlusal line, and sub M2). Furthermore, higher values were also observed for above-normal BMI at the other points, except for the supradental point, where both showed the same averages.	"Males presented thicker facial soft tissues than females; older individuals had higher FSTT values than younger ones; black individuals presented higher means, compared with white individuals, and; subjects with BMI above normal had thicker facial tissues than those with normal BMI."	No	Coordination for the Improvement of Higher Education Personnel - Brazil

Beaini, 2021	Mean FSTT of the face in both sexes.<br><br>Comparison of average soft tissue thickness by sex and age.<br><br>The absolute variation in millimeters among averages of horizontal and vertical facial growth patterns from overall averages.	Most of the difference between men and women was found in the lower portion of the face, with women showing less thickness at all points except the lateral orbital reference point.<br>Age had a minimal quantitative influence and no statistically significant difference in tissue depth measurements.<br>CBCT showed deeper measurements than most necessary tests and other invasive cadaveric tests.<br>Among the means of the different FGPs, there was minimal variation; only the lower sagittal points showed significant differences in the horizontal FGP.<br>The greatest differences occurred between the horizontal malocclusion groups, and differences were found between FSTT Classes II and III.	"The CBCT measuring protocol provides standardization of measurements and is a method to be used in future research. Other advantages of CBCT, such as the subject's up-right position and adjustable volume repositioning, also justify its usage. Limited differences of soft tissue thickness were related to facial growth patterns changes in the vertical or horizontal planes. The Brazilian STT chart, provided by this study, is also a reliable source of data for forensic and clinical purposes."	NR	Academic Cooperation Program in Public Security and Forensic Sciences (CAPES).
Da Silva, 2018*	FSTT according to sex.<br><br>FSTT according to age;<br><br>FSTT according to nutritional status.	This research found three points with greater soft tissue thickness: supra-M2, occlusal line, and sub-M2.<br><br>In this study, the average distances at points such as the lateral orbit, zygomatic arch; supraglenoid; gonion and supra M2 were greater in females than in males. The other average measurements were proportionally higher in males. Statistically significant differences were observed between the sexes ($p < 0.05$) in the distances nasion, rhinion, middle of the labial philtrum, supradentale, and lateral orbit.	"Men had higher averages. There were also significant differences in some distances between age groups. Regarding nutritional status, the distances were smaller among normal weight individuals and greater among obese individuals. When considering different populations, soft tissue thickness showed significant differences in many craniometric points, highlighting how they can be distinct."	No	Foundation for the Support of Science and Technology of Pernambuco
De Almeida, 2013	Differences in soft tissue thickness by sex among Brazilians.<br>Soft tissue thickness reference values among Brazilians..	Only five of the 49 landmarks measured exhibited between-sex differences associated: upper lip margin, superior labial sulcus, stomion, right lateral orbit, and left cheilion.	"This study performed measurements of facial soft tissue thickness among Brazilians. In general, it was observed no differences between sexes and the facial soft tissue depth. This allowed us to construct a unique reference table for both sexes."	NR	NR

Fernandes, 2012	Subject number 4, who is the target subject, was recognized by 26.67% of the 30 examiners, in the reconstruction evaluation carried out using the data from the Brazilian Magnetic Resonance Pattern, by 23.33% in the Brazilian Fresh Cadavers Pattern, and by 20.00% in the International Pattern. In the first two cases, the target subject was the most recognized subject.	It was observed in the International Pattern, the Brazilian Fresh Cadavers Pattern and the Brazilian Magnetic Resonance Pattern, respectively, a correct recognition of 20.00%, 23.33% and 26.67%.<br><br>The correct recognition results for the three facial reconstructions are not statistically different from each other.	"It is not possible to state that one method is better than the other. It would be interesting to repeat this work with a larger sample to compare the results. It was possible to perform the recognition of the forensic digital facial reconstructions of a Brazilian individual using the three facial soft tissue thickness databases used in this research. The correct recognitions of the subject in question indicate that forensic digital facial reconstruction, performed with the parameters used in this study, can be a useful tool."	NR	Coordination for the Improvement of Higher Education Personnel - Brazil
Fernandes, 2013	The target subject was correctly recognized by 40.9% of the examiners in the evaluation of the facial reconstruction created using the data from International Pattern, by 31.8% in the brazilian Magnetic Resonance Pattern and by 31.8% in the Brazilian Fresh Cadavers Pattern. Subject no. 3 also drew a high rate of recognition responses (27.3% in all three patterns). The individuals who composed the face array had a similar facial appearance (all were white Brazilian women, with ages from 33 to 47 years), and subject no. 3 had facial shape and features very close to the target subject.	A pilot protocol proposal was developed to perform 3D digital forensic facial reconstruction using 3ds Max.	"The facial reconstructions without hair were correctly recognized using the three databases of facial soft-tissue thickness. The observed results were higher than the results obtained using facial reconstructions with hair [Fernandes, 2012; Fernandes, 2013], from the same skull, which can indicate that it is better to not use hair, at least when there is no information concerning its characteristics."	NR	Coordination for the Improvement of Higher Education Personnel - Brazil
Fernandes, 2015*	The target subject was recognized by 45.45% of examiners when evaluating the reconstruction made using data from the National Magnetic Resonance Imaging Standard, by 36.36% in the National Fresh Cadaver Standard, and by 45.45% in the International Standard (Table 1). In all cases, it was the most recognized subject.	NA	"The results obtained without hair characterization were better than those obtained in other studies where facial reconstructions with hair were performed and tested. Including hair in facial reconstructions is not recommended, especially when there is no knowledge of, for example, the type, color, or length of the deceased's hair.”	NR	Coordination for the Improvement of Higher Education Personnel - Brazil - Forensic Science

					Program No. 25/2014
Gomes, 2020	Mean of the measurements of craniofacial soft tissue thickness, according to facial type and skeletal classes in the different sexes.	Facial type significantly affected the n-n’, pr-ls’, inc-sto’, sm-sm’, and pg-pg’ regions. The ss-sn’, pr-ls’, inc-sto’, and id-li’ locations were also impacted by skeletal class.<br>Brachycephalic individuals had higher values of soft tissue thickness for pr-ls’ and inc-sto’ than both dolichocephalic and mesocephalic individuals.<br>There is marked sexual dimorphism in soft tissue depth of the craniofacial midline.<br>Class III individuals have thicker soft tissue in regions of the maxilla, while class II subjects present thicker soft tissue in regions of the mandible.	"Skeletal class and facial type influence soft tissue thickness, with a soft tissue compensation in areas with lower skeletal development and/or where bone is positioned more posteriorly. Although the differences in soft tissue thickness may seem numerically low, they reflect exactly the discrepancy between the skeletal bones; therefore, we recommend that specific soft tissue thickness be used for each skeletal class and facial type at the points that differed significantly, and mean values for the points that did not differ, in order to obtain more reliable facial reconstructions in the Brazilian population."	No	Coordination for the Improvement of Higher Education Personnel - Brazil and National Council for Scientific and Technological Development)
Herrera, 2016	Comparison between recognition and similarity rates among reconstructions that used FSTT data obtained from cadavers (two studies), CT, and MRI.	This study showed higher proportions of target recognition through facial approximations constructed with cadaver FSTT datasets compared to medical image datasets.<br><br>The results of the association tests show significant evidence of a higher success rate among men than among women.	It can be concluded that FSTT data obtained through imaging may not facilitate recognition and establish an acceptable level of similarity in Facial Form Analysis (FFA). From the FFA evaluations, it was possible to create a combined dataset, mainly from FSTT datasets for Brazilians, and the greatest gain for the scientific community is the possibility of using the best benefits that each table can offer to achieve a more satisfactory FFA.	NR	NR

Kuhnen, 2021	Means of FSTT measurements, in Brazilian living subadults, according to sex. Differences between the means of males and females. FSTT measurements, in Brazilian living subadults, according to age group. FSTT measurements (in mm), in Brazilian living subadults, according to BMI groups. FSTT measurements , in white and black Brazilian living subadults. FSTT measurements, in Brazilian children, according to sex.	Significant difference between males and females was observed in 9 landmarks: supraglabella, glabella, nasion, rhinion, mid-philtrum, supradentale, infradentale, menton and frontal eminence. In these 9 points, values for males were higher than for females.<br>Comparing children and adolescents, there was a significant difference ($p < 0.05$) at mid-philtrum, infradentale, lateral orbit and zygomatic arch. No significant difference ($p < 0.05$) was observed between white and black subadults.	"This study provides a first set of information for Brazilian subadults – both children and adolescents. It provides FSTT databases of midsagittal and bilateral points, for Brazilian minors. It considers sex, age, ancestry and BMI. The obtained data constitute an important contribution to the realization of FFRs of Brazilian children and adolescents. "	No	Coordination for the Improvement of Higher Education Personnel - Brazil - Finance Code 001.
Miranda, 2018	The positive numbers indicated that the surface of the reconstruction was overestimated in relation to the real face, while the negative numbers indicated that the surface was underestimated. When evaluating each region of the face, it can be observed that in all cases the greatest discrepancies occurred in the cheek and in the eyes that were underestimated, while the chin and the zygomatic area were overestimated.	NA	"The results from this study demonstrate that free open-source software programs are capable of producing 3D computerized forensic craniofacial reconstructions with plausible levels of accuracy and recognition and therefore indicate value for use in forensic investigations. Subsequent studies are required using different tissue thickness and with a larger sample or using alternative software programs to evaluate the method further."	No	NR
Moraes, 2022	"Comparison with the two-dimensional facial approximation performed with composite techniques:<br>Areas of incompatibilities: a) shape of the cranial vault - frontal contour; b)capillary insertion line; c) length of the ears; and d) jawline.<br>Areas of compatibility: a) dolichofacial biotype, with an oval appearance; b) position of the eyebrows; c) position of the eyes; d) height of the ears; e) position of the base of the nose; and f) position of the buccal cupid's bow."	"Comparison with two-dimensional facial approximation performed with digital 3D facial approximation techniques.<br>Areas of incompatibilities: a) brachyfacial biotype; b) capillary insertion line; c)position of the eyebrows; d) height of the ears; and e) jawline.<br>Areas of compatibility: a) shape of the cranial vault – frontal contour; b) position of the eyes; c) position of the base of the nose; and d) Cupid's bow position."	"It is concluded that both Forensic Facial Approximation techniques demonstrated have facial recognition potential. Thus, further research is needed on both Two-dimensional Facial Approximation protocols to assist in the work of forensic professionals in the recognition of unidentified individuals."	No	NR

Moritsugui, 2022	Mean FSTT divided by sex.<br><br>Comparison between soft tissue thickness of Brazilians in the Midwest and Southeast regions.	The male sample exhibited higher FSTT values compared to then female sample, except for the orbital lateral. The major thickness discrepancies between the sexes were found on landmarks the gonion, supra M2, sub M2 and occlusal line.<br><br>A direct relation between FSTT and the aging process can be seen, particularly among females.	"The measurements analyzed in this research, when compared to those collected in the Southeast region of Brazil, are statistically different at many landmarks. However, observing the averages in a specific FSTT chart, indicated that regional differences between the two samples are quantitatively small and they should have minimal influence on facial reconstructions. Therefore, the use of specific FSTT charts is not necessary when performing FFR of individuals from Southeast and Midwest Brazil."	No	NR
Pereira, 2017*	After establishing the biological profile and applying the described methods, manual facial reconstruction was performed, followed by digital reconstruction one month later.	The digital technique was more time-consuming, taking 10 days to complete, while the manual technique was completed in two days.<br>One difficulty that proved unique to the digital technique was the need for prior operator experience. In the manual technique, the operator's practice and artistic skills led to better results, but it was still possible for a lay operator to develop the reconstruction.<br>The operator's greatest difficulty was developing the eyes. In the manual technique, they were disproportionate to the eye socket and eyelids. In the digital technique, the difficulty was rendering the finished face and texturing the eyes at the same time.	"It was concluded that manual facial reconstruction can be performed by a lay operator, with this technique being the most subjective. The digital technique, however, presented greater difficulty due to the operator's lack of experience, resulting in incomplete reconstruction due to the absence of the eyeball."	NR	NR

Pereira, 2021	Was no relationship between the level of knowledge measured according to the group of evaluators, and the correct answer index, once the values were close.	Still regarding correct answers, the average of each group ranged from 20% to 28.75%, being the lowest value observed in group 4 - composed of laymen; and group 3 with the highest average value, composed of undergraduate dentistry students.	The methodology used increased the chances of correct identification by 23.75%, and thus the possibility of finding ante-mortem data for comparison. In other words, this method helps to increase the identification of a missing person. It is important to emphasize that it is an auxiliary tool and cannot be used in isolation and exclusively for identifications. It should be noted that, considering the significant consequences in the lives of the families of unidentified victims, in the social and psychological spheres, as well as in relation to succession, economic and legal issues, the possibility of accuracy in almost 1/4 of the chances makes the tool useful as an auxiliary investigation method.	No	Coordination for the Improvement of Higher Education Personnel - Brazil - Finance Code 001
Pithon, 2014	Mean, maximum and minimum facial soft tissue thickness in children with Class I, II and III (Angle).	With regard to skeletal Class I, the data analysis demonstrated statistical differences ($p < 0.01$) between individuals of the female and male gender only for the means obtained at the rhinion, subnasal and upper lip points.<br>In the evaluation of individuals with malocclusion of Class II and III , no significant difference between the genders was found, with regard to the mean values for the same points evaluated.<br>There were no significant differences among the classes evaluated, except at the stomion, bottom lip and pogonion points. For the stomion, bottom lip and pogonion there was considerable difference between Class II and Class III in both sexes.	"There is no difference in soft tissue thickness among the skeletal Class types evaluated, except between Class II and III for the anthropological points: Stomion, Bottom lip and Pogonion."<br><br>"As regards gender, there was also no statistical difference, except for skeletal Class I in the following anthropological points: rhinion, subnasal and upper lip."	No	No

Rocha, 2003	The quantification of standard error percentage for bone and soft tissue anthropometric measurements,.	3D-CT imaging has been proven to be more accurate in determining measurements than imaging performed directly on CT slices and 2D-CT image reconstruction	"It was possible to establish a qualitative standard for quantitative analysis of 3D-CT images, in bone and soft tissue protocol, with high imaging resolution and processing.<br>3D-CT imaging using the volume rendering technique by computer graphics made it possible to determine the craniofacial measurements with adequate precision."	NR	São Paulo State Research Foundation
Romeiro, 2014	Presentation of forensic facial reconstruction using an automated Hermite Radial Basis Functions model.	The method described in this work produced very accurate results as one may evaluate from the real scanned face of the test subjects. However, the use of a template suitable for gender, age and ethnicity is still required.	"Our novel HRBF detail transfer method for facial reconstruction provides a smooth surface while at the same time preserving the topology of the template mesh. The dissociation between detail and overall shape presented by our deformation method significantly reduces the bias towards the template. Our first informal tests led to successful identification of the subjects. However, broader and more rigorous tests must still be conducted.The automatic reconstruction only takes a few seconds, being a major advantage over any manual method. However, the manual placement of the craniometric points can be time consuming."	NR	Coordination for the Improvement of Higher Education Personnel - Brazil.
Santos, 2008*	Twenty-one points were named, described, and located in multiplanar MRI images.	NA	"It is concluded that establishing the definitions of craniometric points in multiplanar MRI images is an essential step for the correct use of this methodology to obtain data that guarantee objectivity and reliability in the different forensic facial reconstruction techniques for the Brazilian population."	NR	Coordination for the Improvement of Higher Education Personnel - Brazil.

Silvestre, 2024	Relationship of studied measurements between facial skeletal classes with mean values, using ANOVA and Tukey test, and t-test for sex.<br>Relationship of the studied measurements between facial skeletal classes with mean values, using ANOVA and Tukey's test, and t-test for age.<br>Relationship of the studied measurements between gender with mean values, and age with t-test.	Men consistently presented greater soft tissue thickness at various points on the face. Regardless of age, sex, and facial skeletal class, older individuals showed reduced soft tissue thickness in specific areas of the upper lip.<br><br>Soft tissue compensation was observed in areas of less bone development, especially in Class III individuals.	"This study emphasized the significant influence of skeletal class, occlusal pattern, sex, and age on facial soft tissue thickness. Skeletal class plays an important role, along with implications for occlusal pattern, revealing a marked relationship with age and distinct differences between men and women."	NR	NR
Tedeschi-Oliveira, 2009	FSTT means in Brazilians<br><br>Men had greater thicknesses of Facial soft tissues when compared to women in the sample studied.<br><br>Age was not significant.<br><br>The variations related to nutritional status are proportional to the increase of facial fat and do not represent a determining factor in the Facial Reconstruction technique.<br><br>In the sample studied, there was no significant difference between ethnic groups when classified by skin color. However, when compared to results obtained with other populations there were perceived differences.	The comparisons between the measurements from the left and right side of the face showed that the only significant difference was for the Gonion variable. The other left and right side measurements may be considered equal.<br>When the 32 measurements of males and females were compared, values for men were higher, and the differences compared to women were statistically significant for the craniometrical Glabella, mid-philtrum, supramentale, right frontal eminence, left frontal eminence, right SupraOrbita, SupraM2 left, left occlusal line, right SubM2 and left SubM2 points.<br>Regarding the variables related to nutritional status, differences were found for three midline variables and eleven bilateral variables. The values found for the inferior malar, lateral orbit, zygomatic arch, and supraglenoid regions, located in the facial region with highest fat concentration, increased proportionally.<br><br>Showed a statistically significant difference between skin color groups for the variable Nasion and that the different group was the leucoderms group.	"Our study shows that using soft tissue thicknesses found for Americans for Facial Reconstruction in Brazilians will not result in an accurate representation of the individual when alive. Because the Brazilian population is very heterogeneous and mixed, the benchmarks for tables of facial soft tissue thickness obtained by other countries contain differences that must be considered if applied to Facial Reconstruction of Brazilian individuals. We believe that the use of the measurements in our study for Facial Reconstruction in our population will lead to more precise results that will improve the chances of identifying a corpse. This will be of benefit to forensic investigators."	NR	NR

Gomes, 2021	Mean of the linear and angular measurements of the nose according to sex.<br><br>Mean of the linear and angular measurements in the nose, according to skeletal class in males and females.<br><br>Mean of the linear and angular measurements in the nose, according to facial type in males and females.	All linear measurements were larger and statistically significant in men; angular measurements, however, did not differ between the sexes.<br>Regarding the influence of skeletal class on nasal dimensions in men, alterations were observed in both dimensions and angles.<br>In the female group, skeletal class influenced only angular measurements.<br>Regarding facial type, dolichocephalic men presented greater values for length 1 and height than brachycephalic men.<br>Dolichocephalic men also presented greater values for length 2 than mesocephalic men.<br>In women, only one angle differed between facial types, with brachycephalic men presenting greater values than dolichocephalic men.	"The nose linear and angular dimensions of subjects of different sexes, skeletal classes, and facial types by means of CBCT images, and found that all studied factors have some influence on nose morphology. Significantly greater values were found for males in all linear dimensions of the nose, revealing sexual dimorphism. A similarity in some measurements was observed between class I and class II individuals, differing from class III subjects. Nose dimensions also differ between facial types."	No	Coordination for the Improvement of Higher Education Personnel - Brazil and National Council for Scientific and Technological Development
Strapasson, 2017	Mean and standard deviation of the comparison of the studied variables between sexes.<br>Correlation between the studied variables in the external nose and hard tissue.<br>Comparison of the studied variables between different facial typologies.<br>Multiple linear regressions of the relationship between nose width, hard tissue, sex, facial typology, and age.<br>Nasal width can be estimated using the following multiple linear proposition: 23.77 + (c-c x 0.42) + 3.31 (for males).<br>Furthermore, the long face type was associated with nasal width. The male nose was 3.72 mm (average) wider than the female nose.	All measurements differed significantly by sex, always presenting higher values among males.<br>That there were significant, direct, and moderate correlations identified between the upper width of the piriform aperture and the lower width of the piriform aperture and between the nasal width and the distance between the alar insertion points.<br>A correlation was also observed between hard and soft tissue, as there was a significant, direct, and moderate correlation identified between the width of the piriform aperture and the nasal width.<br>The results suggested that for each ten year increase in age, an increase of 0.67 mm in the mean width of the piriform aperture and 0.70 mm in the nasal width was observed.<br>The mean width of the piriform aperture in the group with the short facial type was significantly greater than that observed in the group with the long facial type.	"Identifying relationships between the alar cartilages (nasal wings) and characteristics of the biological profile (traced during the skull anthropological analysis) among subjects from a population little studied thus far. In addition, some parameters (lower width of the piriform aperture, sex, facial type, and age) were found to be associated with the alar curvature, which has been little explored in the literature. Correlations between the nasal width and upper width of the piriform aperture and between lower width of the piriform aperture and distance between the alar insertion points were observed."	NR	Coordination for the Improvement of Higher Education Personnel - Brazil.

		Nasal width could be estimated using the following multiple linear equation: 23.77 + (c-c x 0.42) + 3.31 (for male individuals).			
Strapasson, 2019	Comparison between the actual nasal width and the nasal width estimated by the linear regression equations.<br>Mean of the actual nasal area and nasal area estimated from the hypothesis that the rhi-pn-pr angle is equal to 90° for the whole sample<br>Mean of the real nasal area for the female and male groups, and for the different face types.<br>Mean of the real nasal area according to the age group and face type.	There was a difference of approximately 1 mm between the estimated nasal width and the real nasal width.<br>There was a difference of 101.5 mm2 between the real nasal area and the estimated one. The real nasal area was significantly smaller in the female group when compared to the male group. The nasal area also differed according to the face types, and it was larger in the Long Face group.<br>In addition, for the neutral and Long Face types, the nasal area increased as the age increased.<br>There was no relation of proportion between the nasal width and the variables of piriform aperture width, length of piriform aperture base, and intercanine distance, even considering sex, age group, and facial type.	"The linear regression equations proposed by Strapasson et al. (18) were adequate to estimate nasal width in Brazilian subjects. The data indicate that Long Face type tends to present a straight nasal dorsum."	NR	Coordination for the Improvement of Higher Education Personnel - Brazil - Finance Code 001
Strapasson, 2020	Mean rhinion-pronasale-prosthion angle, according to age range for female and male groups.<br>Difference between actual and estimated distances of actual and estimated pronasale points.	The mean angle studied was 96.5o (± 4.1) for the entire sample, considering a range from 78o to 100o. The female group presented a higher mean of 97.1o (± 3.4) , whereas the male group had a mean angle of 94.5o (± 5.3).<br>The distance between the actual and estimated pn points was measured and resulted in 2.7 (±1.4) mm for the female group and 1.7 mm (± 2.1) for the male group.	"This study validated the technique presented by Tedeschi-Oliveira et al.,24 and established its applicability to a Brazilian sample, with minimum variation. The results prove that a reliable nasal tip may be sculpted using the proposed 90º method."	No	Coordination for the Improvement of Higher Education Personnel - Brazil - Finance Code 001
Tedeschi-Oliveira, 2016	Distribution of the value in degrees of the average nasal projection angle in men and women by age and in the overall average.	For both sexes, the angles varied from 80º to 100º (mean 90º) with a sample composed of younger individuals.<br>The distribution of the angle was different between sexes.<br>It is possible to note that the amplitude (maximum and minimum values) of the intervals is very similar between the	"The analysis of the sample allowed the establishment of an average 908 angle between projections of Prostion and Rhinion points that enable the suggestion of the nasal prominence within 3 mm maximum absolute distance from the real Pronasale point. Those points are easily identifiable on the surface of the skull and, despite a slight statistical difference	No	NR

		sexes; however, the means and medians between these groups were not, due to the sample distribution.	between males and females, it can be applied to most forensic facial reconstruction with satisfactory results."		
Dias, 2016	Mean age in years, linear measurements, and proportions in percentages for height of vermilion borders and width of the mouth, expected value for width; intercanine distance; proportion between intercanine distance and width of the mouth in percentages and divided into age groups for men and women.<br><br>Average age in years and linear measurements in soft and hard tissues, divided by age group, for men and women.<br><br>Proportions between measurements in hard and soft tissues with averages.	The height of the vermilion borders decreased with age in both sexes. The width of men's mouths showed a tendency to increase over time. There was a difference between the sexes, with men having mouths approximately 5 mm wider.The ratio between mental foramina and cheilons in men was approximately 93%, while for women it was 97%, with no differences in verification.	The ratio between the height of the vermilion borders and the width of the mouth showed a tendency to decrease with age in both men and women.<br>Following this trend, in the canine/cheilion ratio, it was observed that, for men, the average for the entire group was 76%. For women, the intercanine distance represented 80% of the mouth width.<br>The length of the path between the incisive foramen and its opening in the nasal fossa showed a ratio equivalent to 1.04 times the height in men and 1.08 times in women.<br>The Philtrum medium and supradentale distance assumed a value of approximately 85% of the height of the vermilion borders. For women, this ratio approached 88%.<br>The infradental-supramental distance for both sexes was equivalent to approximately 55% of the total height.<br>The average height of the lower third of the face compared to the height of the vermilion border of the mouth showed a value approximately 5.4 times greater than the height.	No	No

Fugiwara, 2024	Correspondence between ear length and glabella-subnasale distance.<br><br>Correspondence between length and superior width.<br><br>Nasoaural inclination descriptive data.	In the present study, sexual dimorphism was observed with respect to ear length.<br>Males have longer ears than females. The mean ear length appears to be distinct in different populations. The Brazilian average is 66.39 mm for males and 60.91 mm for females.<br>The parallelism between the ear length and a line that is tangent to the inferior portion of the frontal bone could not be confirmed in most of the study population (65.3%).<br>No parallelism between the ear length (sa'-sba') and the line tangent to the nasal bridge was observed.<br>Considering ear length as the sa'-sba' distance, the angle formed by this line and the Frankfurt plane presented a mean of 81.2o ± 5.14o.<br>No parallelism was observed between sa'-obi' and the nasal bridge (mean 15.2o ± 7.02o), and 23% of cases had an angle lower than 10o.	"The ear placement in the FFR will follow this sequence: (1) Adjust the length values according to sex (66.39 mm for men and 61.91 mm for women). (2) For men, the upper width is 38.25 mm and the lower width is 35.16 mm. For women, 33.91 mm and 31.53 mm, respectively. (3) Regarding the ear position, the skull should be positioned with the Frankfurt Plane parallel to the horizontal plane. Its highest point should coincide with the eyebrow, with a 73° inclination between the Frankfurt Plane and the sa'-obi' line."	No	Coordination for the Improvement of Higher Education Personnel - Brazil - Finance Code 001
