# Supplementary material for: Forensic facial approximation methodologies applied to the Brazilian population: a scoping review
Source: Int J Legal Med. 2026 Aug 8;140(6):3611–21. doi: 10.1007/s00414-026-03947-2 (PMC13658438; doi:10.1007/s00414-026-03947-2)
Supplement: Supplementary file 2 [file 414_2026_3947_MOESM2_ESM.pdf]

Forensic Facial Approximation Methodologies Applied to the Brazilian Population: scoping review

International Journal of Legal Medicine

Paula de Sousa Rachid[1], Daniel Pignatari Mahet Rodrigues[1], Lucas Alves Jural[2], Andrea Vaz Braga Pintor[3], Lucianne Cople Maia[4], Maria Augusta Visconti[4], Andreia Cristina Breda de Souza[4]

[1]DDS, MSc – Faculty of Dentistry of the Universidade Federal do Rio de Janeiro. Rio de Janeiro/RJ – Brazil.

[2]DDS, PhD – Faculty of Dentistry of the Universidade Federal de Uberlândia. Minas Gerais/MG - Brazil.

[3]DDS, MSc, PhD. Faculty of Dentistry of the Universidade Federal Fluminense, Rio de Janeiro, RJ, Brazil.

[4]DDS, MSc, PhD – Faculty of Dentistry of the Universidade Federal do Rio de Janeiro. Rio de Janeiro/RJ – Brazil.

Corresponding author: andreiacrisbreda@gmail.com

Articles excluded after full reading and the reason

Title, Author, Year	Decision	Reason
Facial morphological analysis for the development of a representative atlas of the Brazilian population. Nascimento Falcão, T. 2024	EXCLUDED	It does not present a new, adapted or applied methodology for facial reconstruction, it simply built an atlas.
The nameless dead. Ursin, K. 2007	EXCLUDED	It does not present a new, adapted or applied methodology for facial reconstruction.
