# Supplementary material for: Forensic facial approximation methodologies applied to the Brazilian population: a scoping review
Source: Int J Legal Med. 2026 Aug 8;140(6):3611–21. doi: 10.1007/s00414-026-03947-2 (PMC13658438; doi:10.1007/s00414-026-03947-2)
Supplement: Supplementary file 3 [file 414_2026_3947_MOESM3_ESM.pdf]

Forensic Facial Approximation Methodologies Applied to the Brazilian Population: scoping review

International Journal of Legal Medicine

Paula de Sousa Rachid[1], Daniel Pignatari Mahet Rodrigues[1], Lucas Alves Jural[2], Andrea Vaz Braga Pintor[3], Lucianne Cople Maia[4], Maria Augusta Visconti[4], Andreia Cristina Breda de Souza[4]

[1]DDS, MSc – Faculty of Dentistry of the Universidade Federal do Rio de Janeiro. Rio de Janeiro/RJ – Brazil.

[2]DDS, PhD – Faculty of Dentistry of the Universidade Federal de Uberlândia. Minas Gerais/MG - Brazil.

[3]DDS, MSc, PhD. Faculty of Dentistry of the Universidade Federal Fluminense, Rio de Janeiro, RJ, Brazil.

[4]DDS, MSc, PhD – Faculty of Dentistry of the Universidade Federal do Rio de Janeiro. Rio de Janeiro/RJ – Brazil.

Corresponding author: andreiacrisbreda@gmail.com

Most and least frequent anatomical landmarks found in the studies.

**Anatomic landmarks that appear in more than 50% of the evaluated articles**	**Anatomic landmarks that appear in fewer than 5 of the evaluated articles**	
Glabella<br>Nasion<br>Rhinion<br>Zygion/Zygomatic arch/ Zygomatic<br>Supradentale/Prosthion<br>Supraglenoid<br>Supraglabella<br>Lateral Orbit<br>Occlusal line<br>Mid-philtrum<br>Mid-suborbital / Suborbital / Infraorbital<br>Mid-supraorbital / Supraorbital / medial<br>Supramentale / Chin-lip fold<br>Gonion<br>Supra M2<br>Sub M2	Trichion<br>Bregma<br>Opisthocranium<br>Glabella’<br>Lateral glabella<br>Sellion<br>Nasion’<br>Rhinion’<br>Pronasale<br>Supratip break<br>Alare<br>Alar base<br>Alar curvature<br>Nasal wings<br>Lateral nasal<br>Nasal opening<br>Lateral piriform margin<br>Acanthion / Nasospinale<br>Subnasale<br>Ecthocanthion<br>Endocanthion<br>Mid lateral orbit<br>Medial orbital margin<br>Lateral orbital	Posterior lateral orbital margin<br>Infraorbital foramen<br>Porion<br>Otobasion superius<br>Otobasion inferius<br>Posterotragion<br>Mid masseter<br>Stomion<br>Cheilion<br>Superior labial sulcus<br>Superior central incisor<br>Superior lateral incisor<br>Superior first premolar<br>Superior second premolar<br>Supra-canine / superior canine<br>Infra-canine / sub canina<br>Incision<br>Mid-ramus<br>Mid mandibular<br>Mental tubercule anterior<br>Pogonion’<br>Beneath chin<br>Basion
